# Prevalence and CT angiographic evaluation of coronary artery anomalies in 20,243 consecutive patients: a retrospective study

**DOI:** 10.1186/s43044-026-00744-5

**Published:** 2026-05-19

**Authors:** Seenu Prasanth Adimoulame, Rohan Krishna C R, Rangaraj Ramalingam, Ashita Barthur, Madhav Hegde, Anushree Kumbhalkar, Sruthi Sankar

**Affiliations:** 1 Department of Cardiology, Sri Jayadeva Institute of Cardiovascular Sciences and Research, Bengaluru, India; 2 Heart Institute, Medanta - The Medicity, Gurugram, India; 3 Department of Paediatrics, St. John’s Medical College Hospital, Bengaluru, India

**Keywords:** Coronary artery anomalies, CT angiography, Myocardial bridging, Anomalous origin, Angelini classification, India

## Abstract

**Background:**

Coronary artery anomalies (CAAs) are rare congenital abnormalities involving the origin, course, or structure of coronary arteries. While often incidental, some variants have clinical and procedural implications. With the increasing use of computed tomography coronary angiography (CTCA), more anomalies are being detected, yet large-scale data from India remain limited.

**Objectives:**

To evaluate the prevalence, anatomical patterns, and clinical relevance of CAAs over a 12-year period using CTCA at a high-volume tertiary cardiac center in India.

**Methods:**

In this retrospective study, 20,243 consecutive patients undergoing multidetector CTCA between January 2011 and October 2023 were analyzed. Coronary anomalies were categorized into myocardial bridging (MB) and non-MB types based on Angelini’s classification. Each scan was independently reviewed by both a radiologist and a cardiologist.

**Results:**

Coronary artery anomalies (CAAs) were identified in 1513 patients (overall prevalence 7.5%), largely driven by myocardial bridging (MB). When MB was excluded, the prevalence of non-bridging anomalies was 0.9% (n = 183), including both isolated and combined cases. Isolated myocardial bridging was the most common finding, identified in 1330 patients (87.9%), predominantly involving the mid-segment of the left anterior descending artery. Isolated non-bridging anomalies were observed in 171 patients, while 12 patients had combined MB and non-MB anomalies. Among non-bridging anomalies, the most frequent included anomalous right coronary artery origin from the left sinus with an interarterial course (26.9%), retroaortic left circumflex artery (9.9%), separate origins of the LAD and LCX (8.8%), and high take-off anomalies (7.6%). Most anomalies were classified according to Angelini’s framework, with a small subset remaining unclassified due to atypical anatomical presentations.

**Conclusions:**

This 12-year retrospective cross-sectional study represents one of the largest single-center CTCA-based datasets on coronary artery anomalies (CAAs) globally. The findings highlight the utility of CTCA in detecting and characterizing both benign and potentially significant anomalies. The anatomical insights derived from this cohort have direct clinical relevance, aiding interventional cardiologists in procedural planning and risk stratification. Future multicenter studies are warranted to further refine diagnostic algorithms and management strategies across diverse populations.

**Supplementary Information:**

The online version contains supplementary material available at 10.1186/s43044-026-00744-5.

## Introduction

Coronary artery anomalies (CAAs) represent diverse congenital abnormalities affecting the origin, course, or structure of coronary arteries. While frequently incidental, certain variants carry significant clinical and procedural implications. The advent of multidetector computed tomography coronary angiography (CTCA) has markedly enhanced non-invasive detection and anatomical characterization of CAAs due to superior spatial resolution compared to traditional imaging modalities [1, 2].

The reported prevalence of CAAs varies significantly by population and diagnostic technique, ranging from approximately 0.17% in autopsy studies to about 1.2% in invasive angiography [3, 4]. However, CTCA-based studies, particularly from India, have demonstrated substantially higher detection rates—up to 10.09% in some cohorts—highlighting the enhanced sensitivity of modern imaging techniques [4]. Variants such as anomalous coronary artery origin from the opposite sinus (ACAOS) and myocardial bridging are clinically important, linked to myocardial ischemia, arrhythmias, and rarely sudden cardiac death, especially among younger populations and athletes [5, 6]. These anomalies can also complicate catheter engagement and procedural interventions, underscoring their significance for interventional cardiologists.

Angelini and colleagues developed a widely adopted classification that categorizes CAAs into anomalies of origin and course, intrinsic coronary arterial anatomy, termination, and anomalous anastomotic vessels, excluding normal variants [1]. The system excludes normal variants and defines anomalies based on a population prevalence of less than 1% [1]. Despite increased global awareness, large-scale Indian data remain sparse, predominantly comprising small series or autopsy-based studies. India's substantial genetic diversity and unique cardiovascular profile, characterized by high burdens of premature coronary artery disease, diabetes, and diverse lifestyle-related risks, necessitate tailored epidemiological insights. The present study addresses this knowledge gap, systematically analyzing CAAs using Angelini’s classification in over 20,000 CTCA scans from a high-volume Indian tertiary cardiac centre over 12 years, providing vital anatomical and procedural insights specific to the Indian context.

## Materials and methods

### Study design and population

This retrospective cross-sectional study was conducted at Sri Jayadeva Institute of Cardiovascular Sciences and Research, Bengaluru—a high-volume tertiary cardiac centre. A total of 20,243 consecutive patients who underwent computed tomography coronary angiography (CTCA) between January 2011 and October 2023 were included. The Institutional Ethics Committee approved the study protocol, and informed consent was waived owing to the retrospective nature of the analysis.

### Eligibility criteria

#### Inclusion criteria:

All patients undergoing CTCA during the study period (January 2011 to October 2023)

#### Exclusion criteria

- Prior coronary artery bypass grafting (CABG)
- Severe coronary calcification impeding image interpretation
- Uncontrolled arrhythmias or resting heart rate > 80 bpm
- Known allergy to iodinated contrast agents
- Renal dysfunction (serum creatinine > 3.0 mg/dL)
- Pregnancy
- Clinically significant respiratory compromise, as judged by the treating physician

### CT angiography acquisition protocol

Scans were performed using a 64-slice Philips Brilliance scanner (Best, Netherlands) (2011–January 2022) and a 128-slice GE Revolution Maxima system (Milwaukee, USA) (February 2022–October 2023). Patients received a sublingual tablet of isosorbide dinitrate (5 mg) before contrast administration. In patients with a resting heart rate > 65/min, oral metoprolol tartrate 5 mg was administered 1.5 h before scanning or intravenous metoprolol tartrate 5 mg during the procedure.

### Scanning parameters

- 100–120 kV, Auto mA 300—500
- Rotation time 0.35 s
- Detector width of 40 mm
- 0.625 mm slice thickness, 0.625 mm increment

### Contrast protocol

- Nonionic iodinated contrast (Iohexol – OMNIPAQUE 350 mg/mL) at 1.5 mL/kg body weight at the rate of 5 mL/s followed by
- 30 mL saline flush
- Automated bolus tracking with threshold set at 140–160 HU in the ascending aorta

### Image reconstruction and postprocessing

Reconstruction was performed at 70%, 75%, and 80% of the R–R interval. Curved multiplanar reformats and 3D volume-rendered images were created. All studies were independently reviewed by an experienced radiologist and cardiologist. Discrepancies were resolved by consensus.

### Data analysis and classification

All CTCA datasets were independently reviewed by an experienced radiologist and cardiologist, with discrepancies resolved by consensus; interobserver variability was not formally quantified. Coronary anomalies were classified using a dual framework capturing both patient-level prevalence and anatomical diversity. Myocardial bridging was considered a commonly reported anatomical variant and analyzed separately from non-bridging anomalies. At the patient level, anomalies were grouped as myocardial bridging, non-bridging, or combined. Individual anomalies were further categorized according to Angelini’s classification. As some patients had more than one anomaly, the total number of anomalies (n = 1525) exceeded the number of patients with anomalies (n = 1513). Continuous variables are presented as mean ± standard deviation, and categorical variables as frequencies and percentages.

## Results

**Fig. 1:**
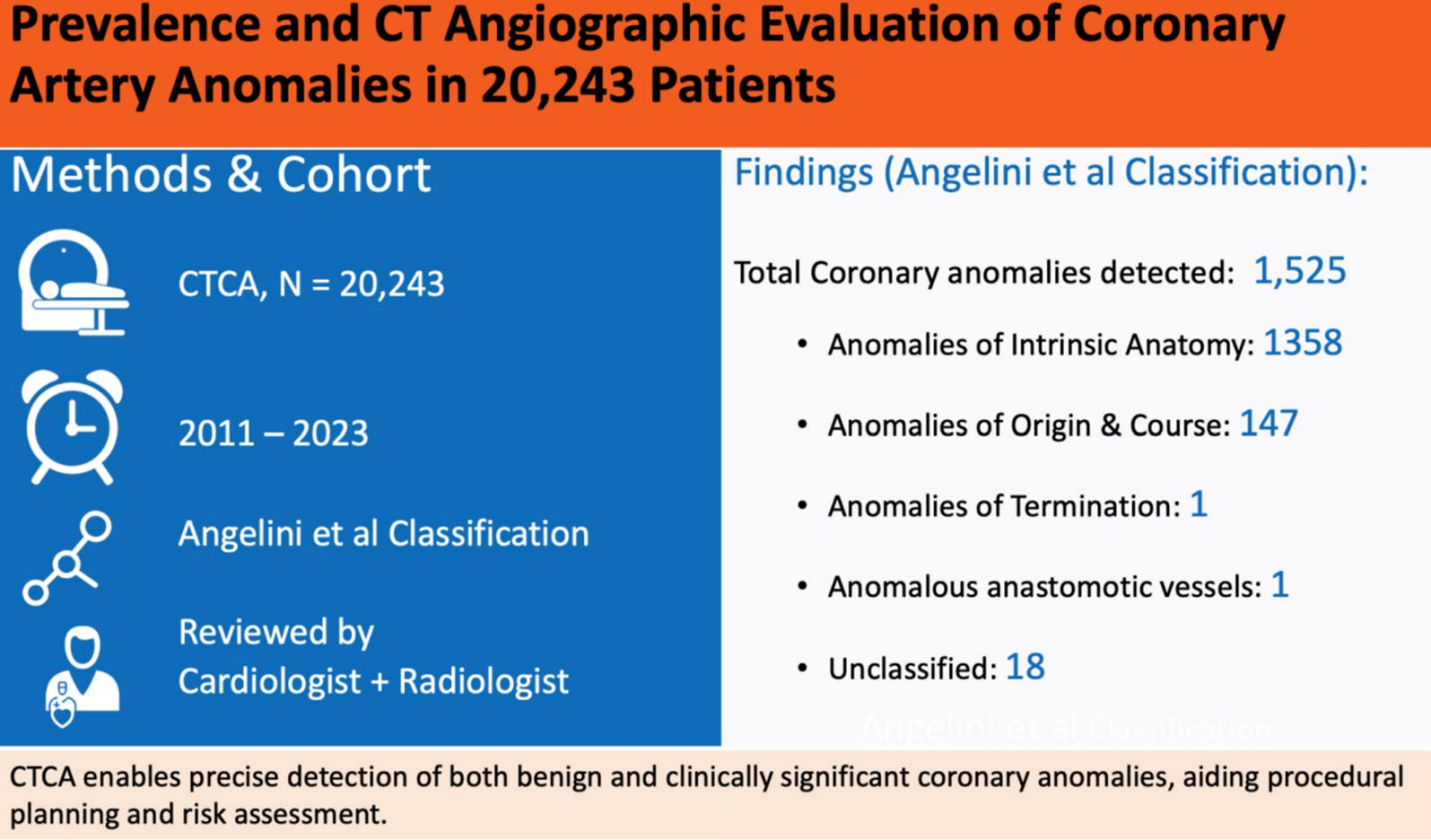
Outline of the study

Coronary Artery Anomalies: Between January 2011 and October 2023, 20,243 patients underwent coronary computed tomography angiography (CTCA). Scans utilized a 64-slice Philips Brilliance CT system (2011–January 2022) and subsequently a 128-slice GE Revolution Maxima system (February 2022–October 2023). Coronary artery anomalies (CAAs) were identified in 1,513 patients (prevalence 7.5%), largely driven by myocardial bridging. As some patients had more than one anomaly, the total number of anomalies was 1525. When myocardial bridging was excluded, the prevalence of non-bridging anomalies was 0.9% (n = 183). The outline of the study is presented in Fig. 1.

**Fig. 2:**
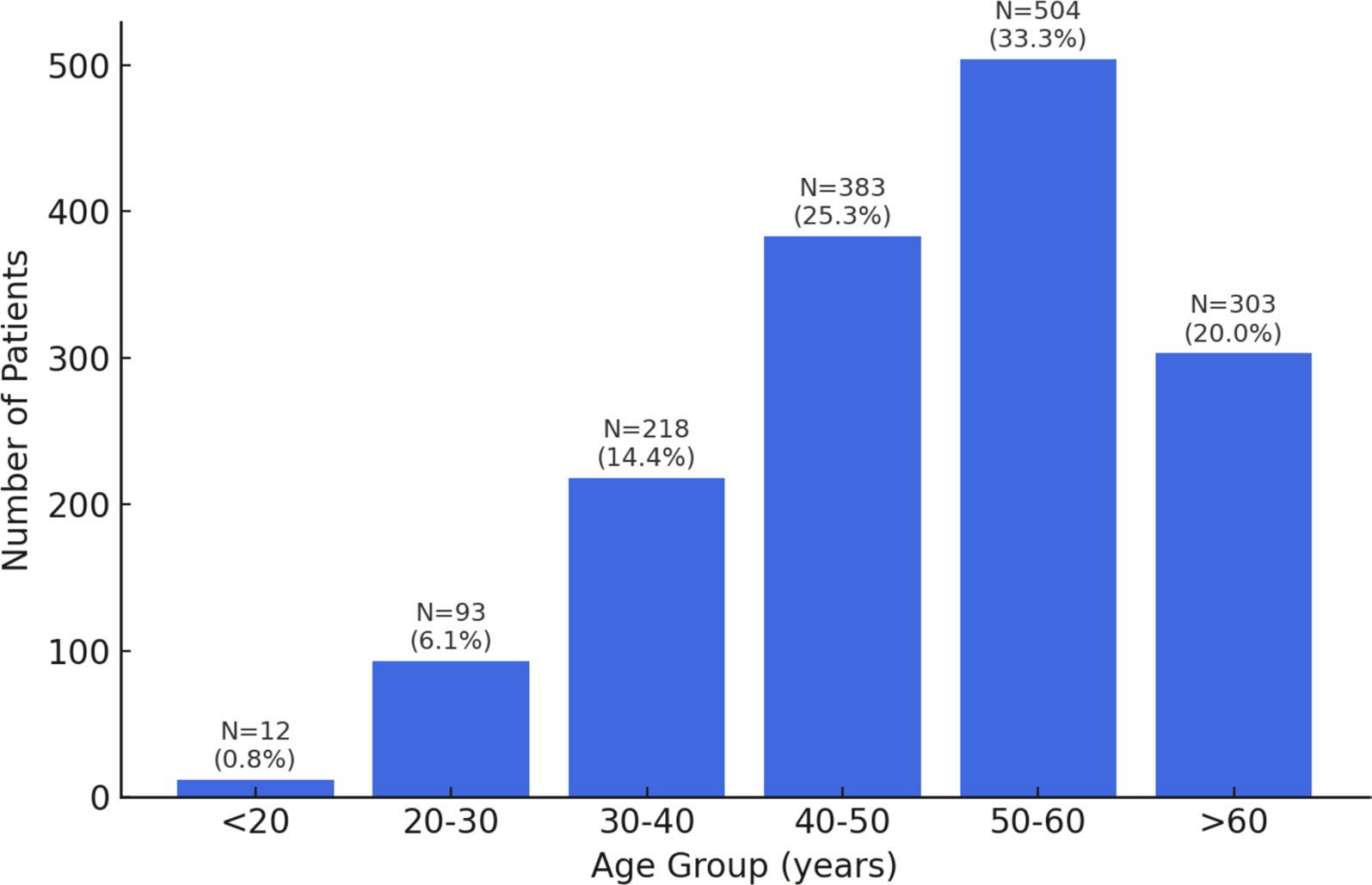
Age-wise distribution of coronary artery anomalies (CAAs) detected on CTCA, showing the number (N) and percentage (%) of patients in each age group

Patient Demographics: Of the 1513 patients with CAAs, 1029 (68%) were male, and 483 (32%) female. Mean age was 49 ± 11 years (range, 13–80 years). Age-wise distribution is presented in Fig. 2.

Types of Coronary Artery Anomalies: Isolated myocardial bridging was the most common anomaly, found in 1330 patients (87.9%), predominantly involving the left anterior descending artery (LAD). Non-bridging anomalies were identified in 183 patients (12.1% of all anomalies), including 171 isolated cases and 12 cases with coexisting myocardial bridging.

**Fig. 3:**
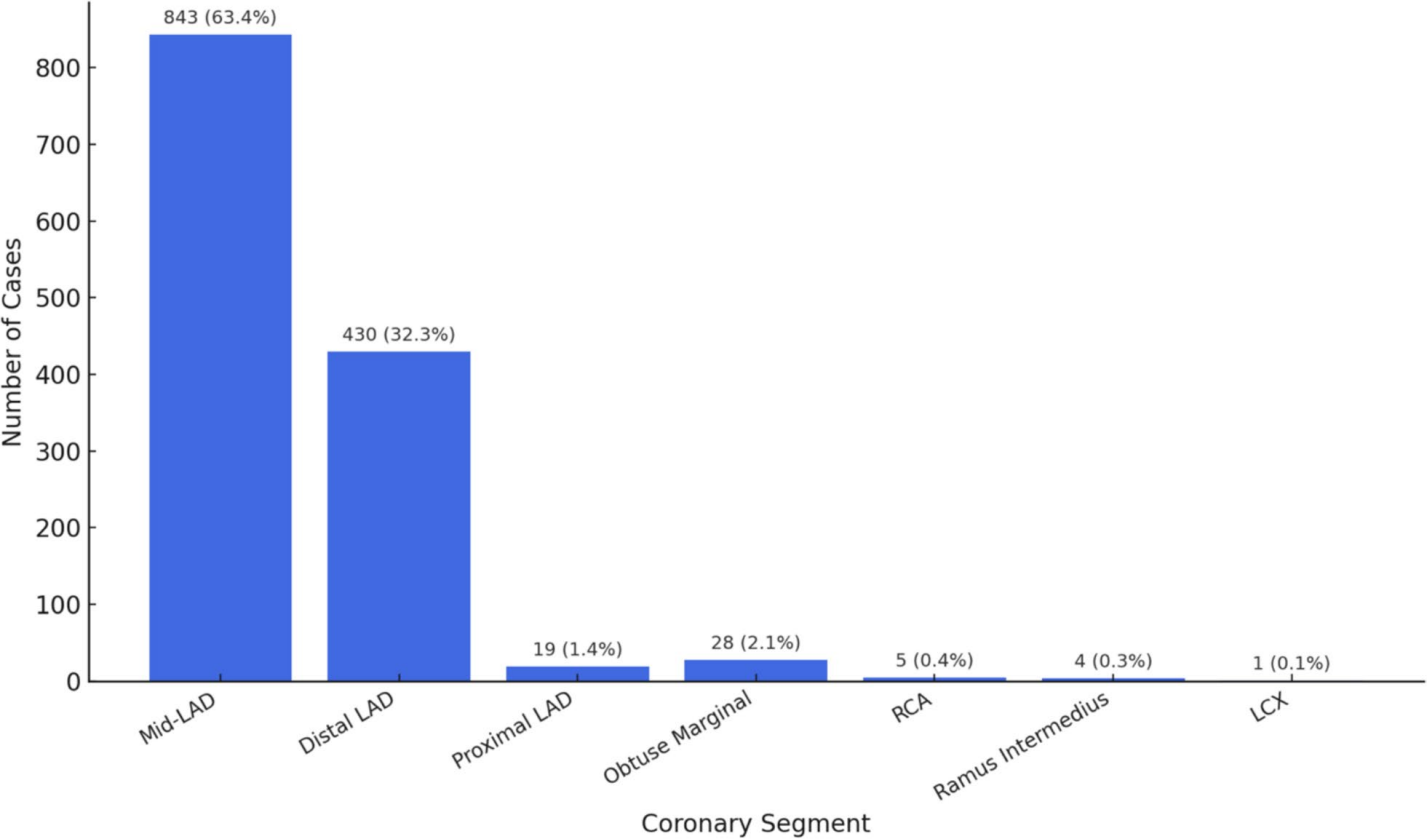
Segmental distribution of myocardial bridging (n = 1330)

Isolated Myocardial Bridging: Of 1330 isolated myocardial bridging cases, the LAD was involved in 1304 cases (98.0%): mid-LAD in 843 (63.4%), distal LAD in 430 (32.3%), and proximal LAD in 19 (1.4%). Less commonly involved vessels included obtuse marginal branch (n = 28; 2.1%), right coronary artery (RCA; n = 5; 0.4%), ramus intermedius (n = 4; 0.3%), and left circumflex artery (LCX; n = 1; 0.1%) (Fig. 3).

**Fig. 4:**
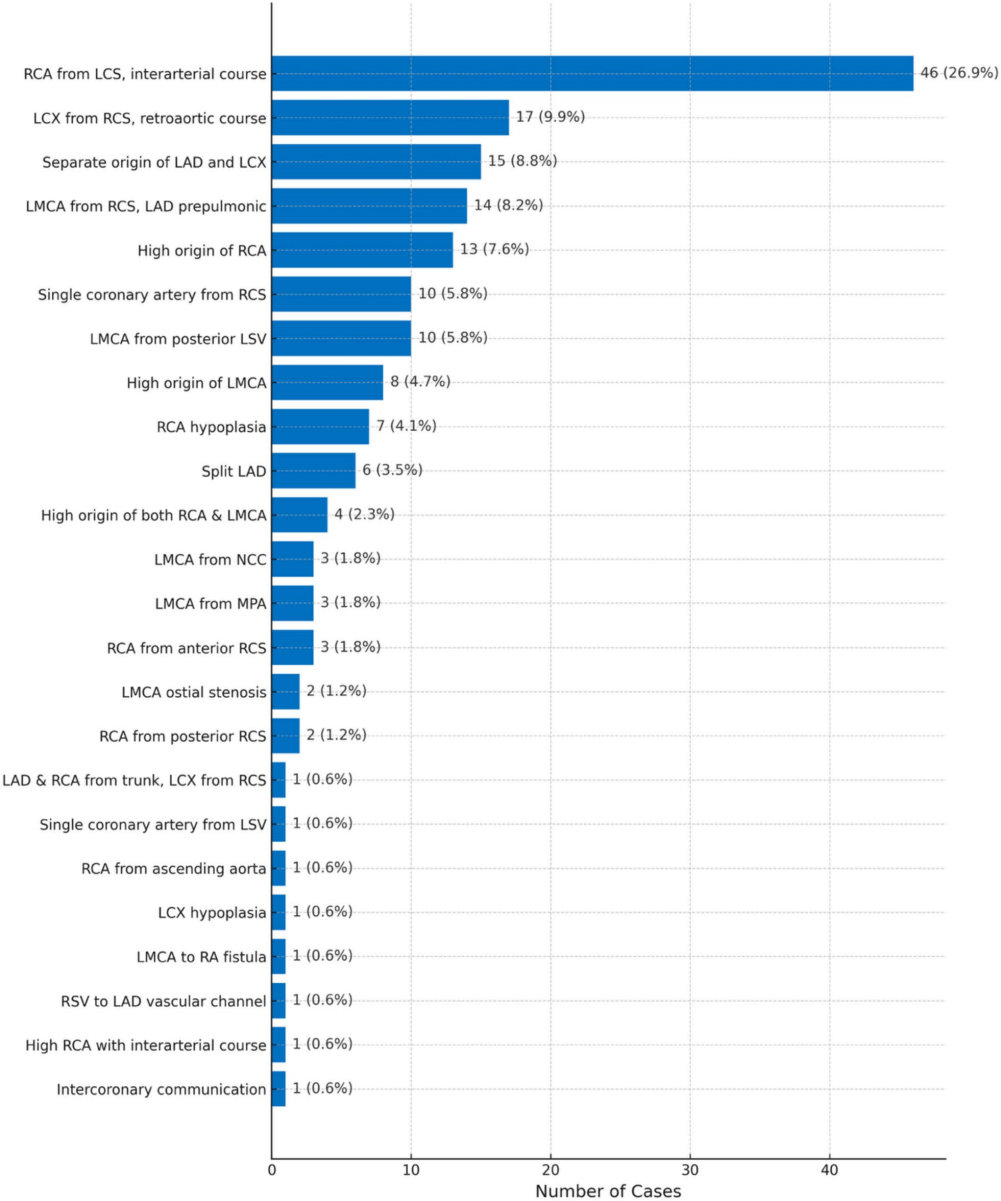
Distribution of coronary artery anomalies other than myocardial bridging (n = 171), arranged in the order of frequency

Non-bridging Coronary Artery Anomalies: Among 171 non-MB anomalies, RCA originating from left coronary sinus (LCS) with an interarterial course was most prevalent (n = 46; 26.9%). Other variants included LCX from right coronary sinus (RCS) with retroaortic course (n = 17; 9.9%), separate LAD and LCX origins (n = 15; 8.8%), and prepulmonic LMCA from RCS (n = 14; 8.2%). Posterior LCS LMCA origin was found in 10 patients (5.8%), single coronary arteries arose from RCS (n = 10; 5.8%) and LCS (n = 1; 0.6%), and high take-offs included RCA (n = 13; 7.6%), LMCA (n = 8; 4.7%), and both RCA and LMCA combined (n = 4; 2.3%). Rare anomalies included LMCA from non-coronary cusp (n = 3), LMCA from pulmonary artery (n = 3), RCA from ascending aorta (n = 1), RCA hypoplasia (n = 7), LCX hypoplasia (n = 1), LMCA ostial stenosis (n = 2), split LAD (n = 6), LMCA-to-RA fistula (n = 1), and intercoronary communication (n = 1). Additional rare variants included RCA from anterior (n = 3) and posterior RCS (n = 2), and accessory vascular channel from RCS to LAD (n = 1). Full distribution is shown in Fig. 4.

Combined Myocardial Bridging and Other Coronary Anomalies: Combined anomalies occurred in 12 patients (0.8%). Most common combinations were RCA from LCS with interarterial course (n = 6), separate LAD/LCX origins (n = 3), and LMCA from posterior LCS (n = 3). Bridging involved mid-LAD (n = 6) and distal LAD (n = 6).

Angelini Classification: All the coronary anomalies were classified using Angelini’s schema (Table 1). Most anomalies fit predefined categories, except for a small subset (RCA from anterior or posterior RCS, LMCA from posterior LCS) that remained unclassified yet potentially clinically significant.

**Table 1:** Angelini classification of coronary artery anomalies

Classification and anomaly description	N	%
*A: Anomalies of origin and course*	**147**	
1. Absent left main trunk (LMCA) with separate origins of LAD and LCX	18	1.2
2. Anomalous location of coronary ostium within aortic root or near proper coronary cusp:	26	
a. High origin of RCA above STJ	14	0.9
b. High origin of LMCA above STJ	8	0.5
c. High origin of both RCA & LMCA above STJ	4	0.3
3. Anomalous location of coronary ostium outside normal coronary cusp:	4	
a. RCA from ascending aorta	1	0.1
b. LMCA from main pulmonary artery	3	0.2
4. Anomalous location of coronary ostium at improper sinus:	88	
a. RCA from LCS, with anomalous course	52	3.4
b. LAD from RCS	1	0.1
c. LCX from RCS	17	1.1
d. LMCA from RCS (pre-pulmonic course)	15	1.0
e. LMCA from NCS, with anomalous course	3	0.2
5. Single coronary artery (SCA):	11	
a. From RCS	10	0.7
b. From LCS	1	0.1
*B: Anomalies of intrinsic anatomy*	**1358**	
1. Congenital ostial stenosis – LMCA	2	0.1
2. Coronary hypoplasia – RCA	7	0.5
3. Coronary hypoplasia – LCX	1	0.1
4. Intramural coronary artery (Muscular bridging)	1342	88.0
5. Split LAD	6	0.4
*C: Anomalies of termination*	**1**	
1. Fistula from LMCA to right atrium	1	0.1
D: Anomalous anastomotic vessels	**1**	
1. Intercoronary communication	1	0.1
*E. Unclassified*	**18**	
1. RCA from anterior RCS	3	0.2
2. RCA from posterior RCS	2	0.1
3. LMCA from posterior LCS	13	0.9
*Total*	**1525**	**100**

Classification of coronary artery anomalies (n = 1525) as per Angelini’s system, including 1330 isolated myocardial bridging cases, 171 non-bridging anomalies, and 12 combined cases. Frequencies (n) and percentages are based on the total number of anomalies (n = 1525).

## Discussion

In this large single-center CTCA study of 20,243 patients, the overall prevalence of coronary artery anomalies (CAAs) was 7.5%, with 1,525 anomalies identified due to multiple anomalies in some patients. This is higher than invasive coronary angiography (ICA)–based reports (0.6–2.1%) [3, 7], reflecting the superior sensitivity of CTCA in delineating coronary anatomy.

Our findings are consistent with prior CTCA-based studies. Among Indian cohorts, Sirasapalli et al. reported a prevalence of 10.09% [4], while Singh et al. observed 9.6% [8], with additional variability noted by Ganga et al. [9]. International studies have demonstrated comparable detection rates, including 1.16% reported by Namgung and Kim [10] and 2.0% by Ghadri et al. [11]. Notably, when myocardial bridging was excluded, the prevalence of non-bridging anomalies in our cohort was 0.9% (n = 183), closely aligning with the findings of Kothari et al. [12]. These variations likely reflect differences in population characteristics, imaging techniques, and classification criteria, with Angelini’s framework providing a standardized basis for reporting [1, 2].

The observed male predominance (68%) and mean age (49 years) are consistent with prior reports, reflecting typical adult presentation patterns [11, 13].

Myocardial bridging (MB) was the most frequent finding (6.6%), predominantly involving the LAD, in line with CTCA-based studies. However, reported prevalence varies widely, ranging from 9.4 to 28.4% across different populations [14–16], likely due to differences in imaging techniques, diagnostic criteria, and reporting thresholds. In our cohort, 12 patients (0.8%) demonstrated coexistent MB and another anomaly, most commonly an interarterial RCA from the left sinus, followed by separate LAD and LCX origins and anomalous LMCA origin. Although uncommon, such combined anomalies have been described and highlight the importance of comprehensive CTCA evaluation [17].

Among the 183 patients with non-MB coronary anomalies, the most frequent were anomalous origin of the right coronary artery (RCA) from the left sinus with an interarterial course (24.7%), followed by separate origins of the LAD and LCX (9.9%) and retroaortic LCX (9.3%), consistent with prior CTCA-based studies [4, 18, 19].

Anomalous origin from the opposite sinus with an interarterial course represents a clinically important high-risk subset associated with myocardial ischemia and sudden cardiac death [6, 20], accounting for 52.2% of non-MB anomalies in our cohort. In contrast, anomalies such as retroaortic LCX are generally benign and clinically silent [21].

Additional anomalies included high take-off (14.2%), single coronary artery (6.5%), and anomalous origin from non-coronary sinuses or the pulmonary artery (3.8%), including three cases of ALCAPA [3, 22]. Intrinsic coronary abnormalities were rare (0.1%), most commonly RCA/LCX hypoplasia and LMCA ostial stenosis, both of which may have significant clinical implications [23]. Split LAD and other rare anomalies, including coronary cameral fistula and intercoronary communication, were infrequent but may pose diagnostic and procedural challenges [24, 25].

## Anomalous coronary origins: recognition and interventional relevance

**Fig. 5:**
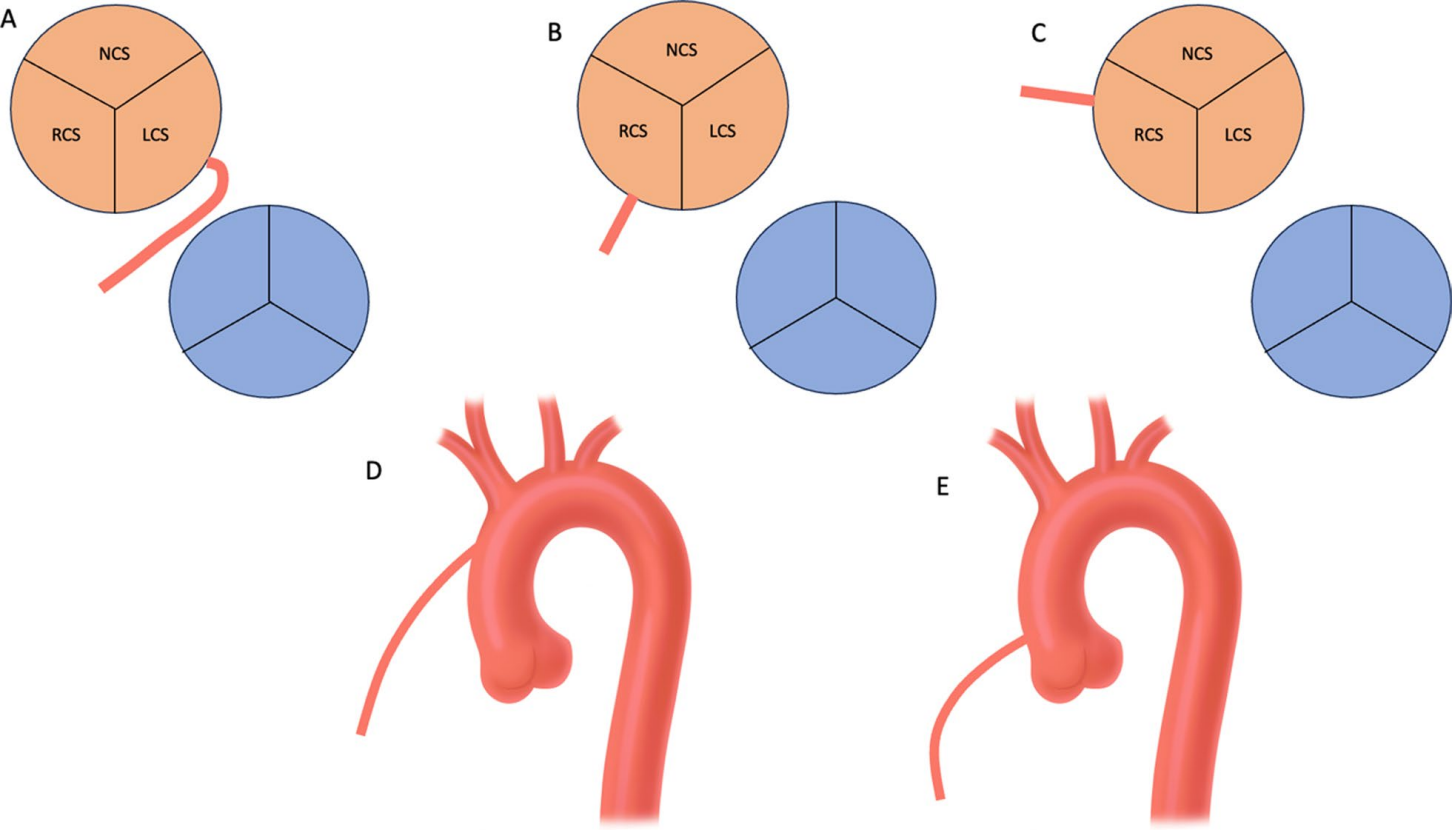
Schematic illustration of anomalous RCA origins. **A** – RCA origin from the left coronary sinus (interarterial course). **B** – RCA origin from anterior aspect of right sinus of Valsalva. **C** – RCA origin from posterior aspect of right sinus of Valsalva. **D** – RCA origin from ascending aorta. **E** – High origin of RCA above the sinotubular junction. *LCS* – Left coronary sinus; *RCS* – Right coronary sinus; *NCS* – Non-coronary sinus

Anomalous coronary origins represent a clinically important subset of CAAs with direct procedural implications. In our cohort, anomalous origin of the right coronary artery (RCA) was identified in 76 patients (5.0%), most commonly arising from the left sinus with an interarterial course (n = 52), followed by high take-off variants (n = 18). Less frequent origins included atypical positions within the right sinus and, rarely, from the ascending aorta (Fig. 5). These configurations may significantly hinder catheter engagement and selective cannulation, particularly in urgent settings [1, 3, 20, 26, 27].

**Fig. 6:**
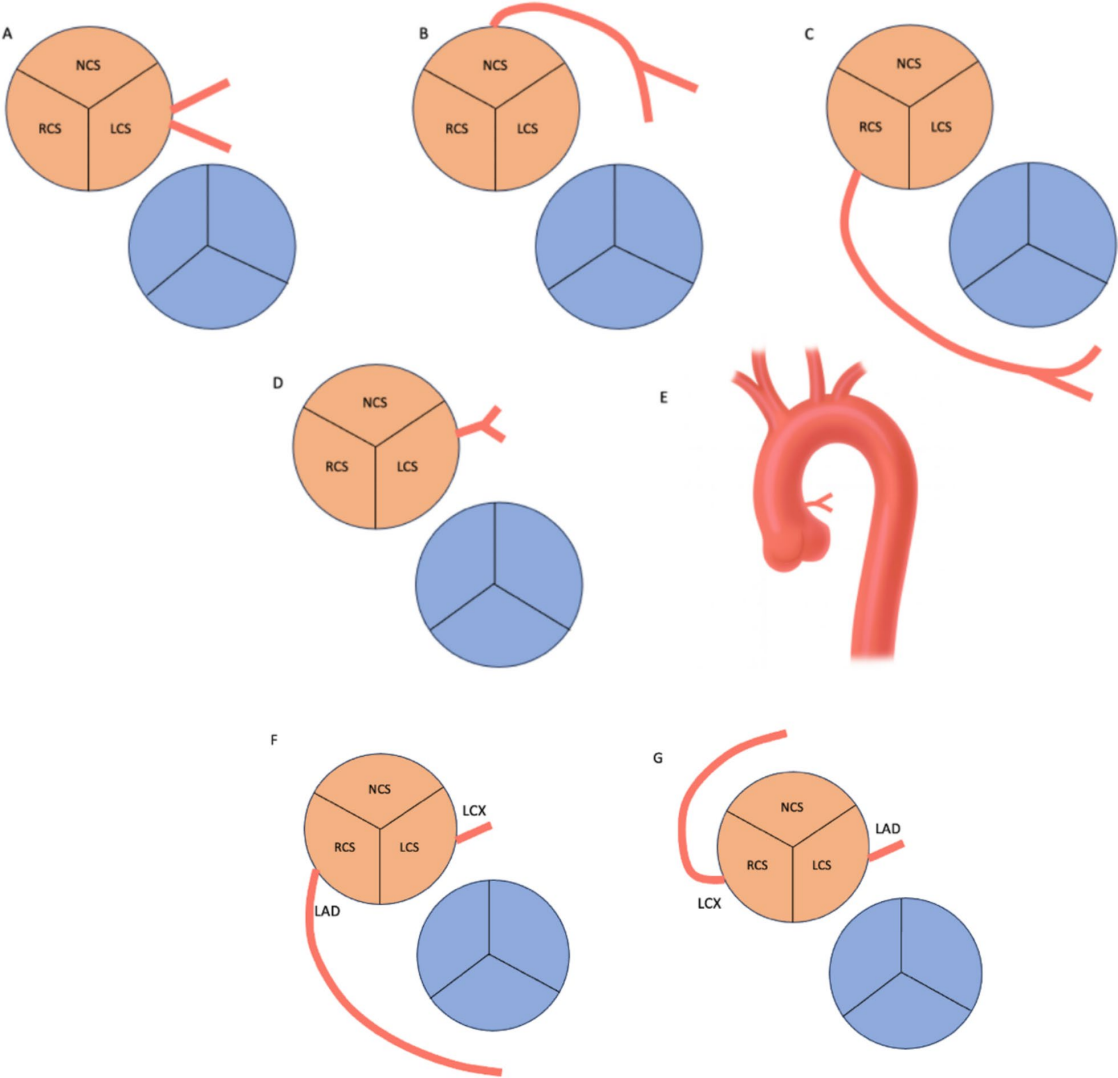
Schematic illustration of anomalous origins of the left coronary arterial system. **A –** Absent left main trunk (LMCA) with separate origins of the LAD and LCX. **B –**LMCA originating from the non-coronary sinus (NCS). **C –**LMCA arising from the right coronary sinus (RCS) with a pre-pulmonic course. **D –** LMCA originating from the posterior left sinus of Valsalva (LCS). **E –** High origin of the LMCA above the sinotubular junction (STJ). **F –** LAD arising from the RCS with a pre-pulmonic course. **G –** LCX arising from the RCS with a retroaortic course. *LCS* – Left coronary sinus; *RCS* – Right coronary sinus; *NCS* – Non-coronary sinus

Similarly, anomalies involving the left coronary system were observed in 76 patients (5.0%), most commonly as absent left main trunk with separate LAD and LCX origins (n = 18), followed by anomalous LMCA origin from the right sinus (n = 15) and high take-off variants (n = 12). These variations may alter ostial location, angulation, and vessel course, increasing technical complexity during coronary angiography and intervention (Fig. 6) [28–30].

The detailed anatomical insights derived from this large CTCA dataset provide important clinical and procedural relevance, particularly for interventional cardiologists encountering anomalous coronary origins. Pre-procedural CTCA may aid in catheter selection and procedural planning by accurately delineating coronary anatomy [18, 21, 27, 30, 31].

However, the absence of functional or ischemic correlation remains an important limitation, as this intermediate-risk cohort was assessed using anatomical imaging alone. This should be considered when interpreting the clinical relevance of the findings.

Strengths: This study represents one of the largest single-center CTCA-based studies of coronary artery anomalies globally, providing robust epidemiological data. The standardized classification and systematic image interpretation enhance diagnostic accuracy, offering critical anatomical and clinical insights relevant to interventional practice.

Limitations: This study is limited by the absence of functional or ischemic correlation, as the analysis was based solely on anatomical imaging, precluding direct assessment of the clinical significance of identified anomalies. Additionally, the study population comprised an intermediate-risk cohort referred for CTCA based on clinical indications, which may introduce referral bias and limit generalizability. The single-center, tertiary care setting may further restrict broader applicability. While the analysis was primarily descriptive, reflecting the study’s focus on large-scale anatomical characterization, future studies incorporating subgroup analyses, temporal trends, and clinical outcomes may provide additional insights.

## Conclusion

This 12-year retrospective study represents one of the largest single-center CTCA-based datasets on coronary artery anomalies from India. While the overall prevalence (7.5%) was largely driven by myocardial bridging, clinically relevant non-bridging anomalies constituted a smaller subset (0.9%) and aligned with prior CTCA-based studies.

Beyond prevalence, this study provides comprehensive anatomical characterization of CAAs, with implications for procedural planning in interventional cardiology. Although derived from an intermediate-risk population without functional correlation, these findings offer valuable epidemiological and anatomical insights. Future multicenter studies incorporating clinical and functional outcomes are warranted.

## Supplementary Information

Additional file 1 (DOCX 1861 KB)

Additional file 2 (MP4 2865 KB)

Additional file 3 (MP4 2777 KB)

## Data Availability

The datasets generated and/or analysed during the current study are available from the corresponding author upon reasonable request.

## Abbreviations

ACAOS: Anomalous coronary artery origin from the opposite sinus
ALCAPA: Anomalous left coronary artery from the pulmonary artery
Bpm: Beats per minute
CABG: Coronary artery bypass grafting
CAD: Coronary artery disease
CAA: Coronary artery anomaly
CAAs: Coronary artery anomalies
CTCA: Computed tomography coronary angiography
ECG: Electrocardiogram
HU: Hounsfield unit
LAD: Left anterior descending (artery)
LCX: Left circumflex (artery)
LMCA: Left main coronary artery
LCS: Left coronary sinus
MB: Myocardial bridging
MDCT-CA: Multidetector computed tomography coronary angiography
NCS: Non-coronary sinus
OM: Obtuse marginal (branch)
RCA: Right coronary artery
RCS: Right coronary sinus
RI: Ramus intermedius
SCA: Single coronary artery
STJ: Sinotubular junction
