# Supplementary material for: Prevalence and CT angiographic evaluation of coronary artery anomalies in 20,243 consecutive patients: a retrospective study
Source: Egypt Heart J. 2026 May 19;78:35. doi: 10.1186/s43044-026-00744-5 (PMC13187100; doi:10.1186/s43044-026-00744-5)
Supplement: Supplementary file 1 — Additional file 1 (DOCX 1861 KB) [file 43044_2026_744_MOESM1_ESM.docx]

**Supplementary:**

**Figure 7.** CT showing a normal RCA origin with a dilated LMCA giving rise to the LAD and LCX, and a coronary–cameral fistula draining into the right atrium (Video 1).

**
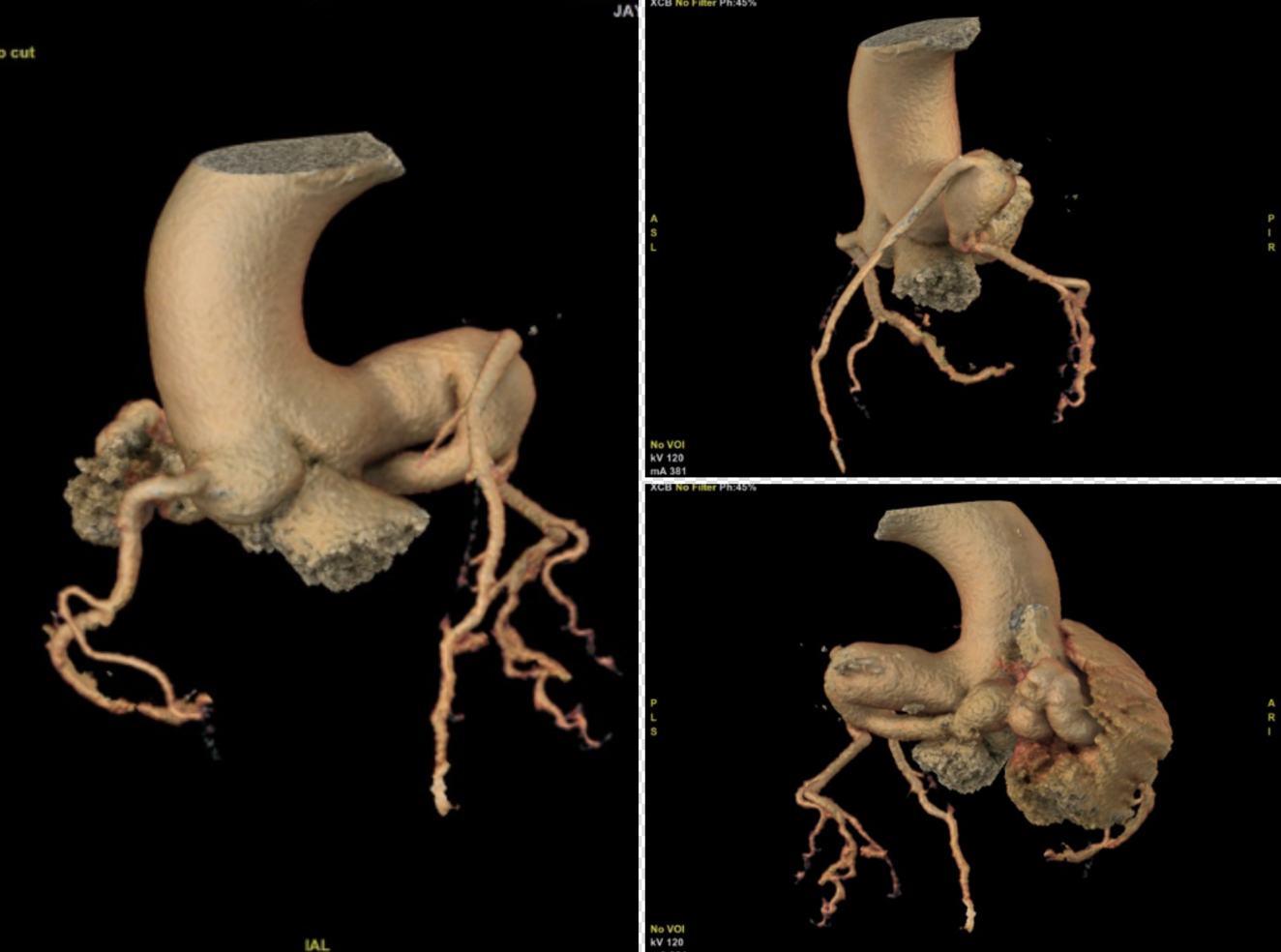
**

LCX

LAD

RCA

RCA

LCX

LAD

C

B

A

**Figure 8.** CT showing a single coronary artery giving rise to the RCA and dual LAD; the short LAD has a pre-pulmonic course, and the long LAD has a transeptal course. The LCX continues as the major OM (Video 2).

**
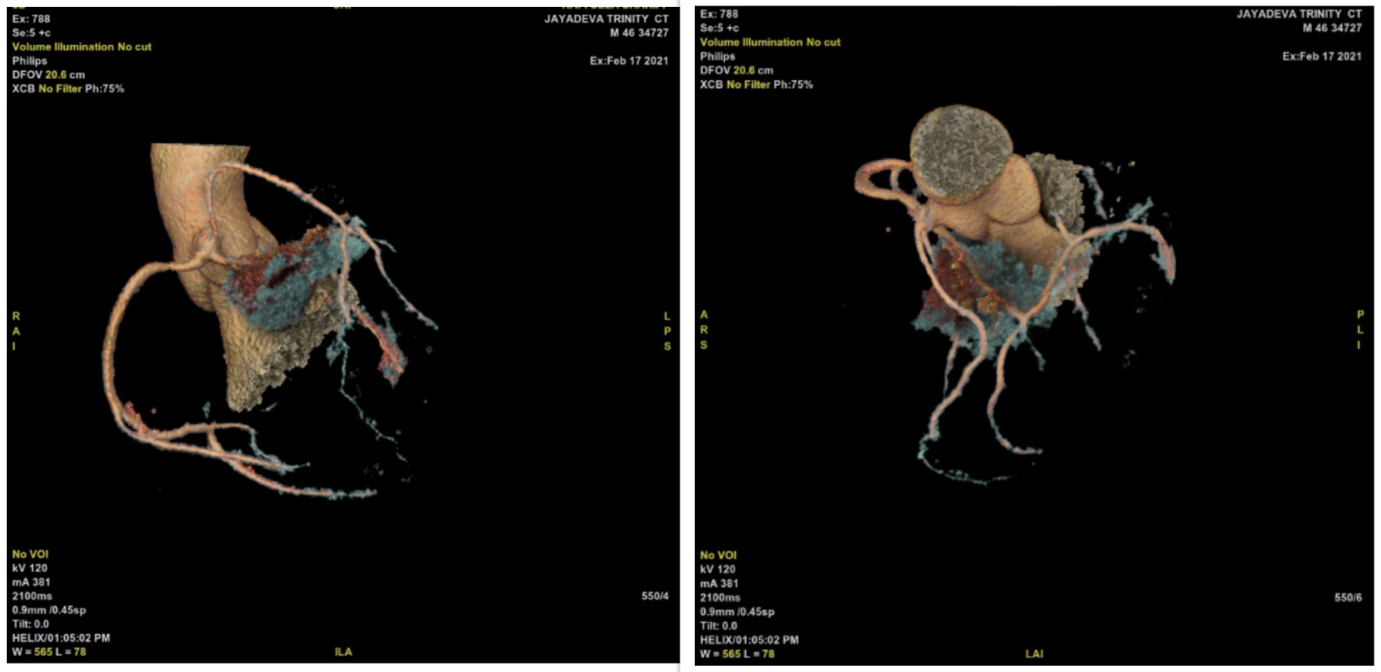
**

Long LAD

Short LAD

RCA

B

A

**Figure 9.** CT showing separate origin of LCX from the right sinus of Valsalva with a retroaortic course. Mid-LCX compression (double arrow) in systole is associated with inferolateral wall thinning and infarction (arrow).

**
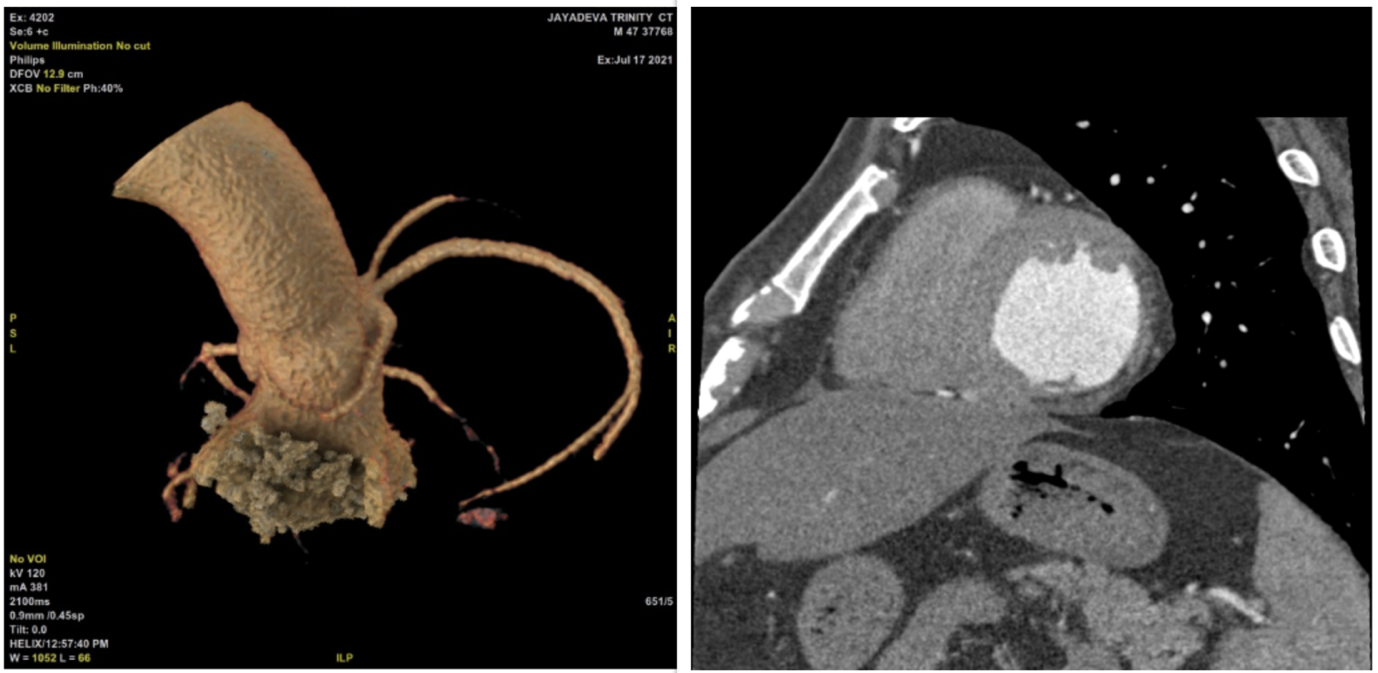
**

RCA

LCX

LAD

B

A
